# Increased Expression of VEGF and CD31 in Postradiation Rectal Tissue: Implications for Radiation Proctitis

**DOI:** 10.1155/2013/515048

**Published:** 2013-05-08

**Authors:** G. Karamanolis, I. Delladetsima, V. Kouloulias, K. Papaxoinis, I. Panayiotides, D. Haldeopoulos, K. Triantafyllou, N. Kelekis, S. D. Ladas

**Affiliations:** ^1^Hepatogastroenterology Unit, 1st Department of Internal Medicine—Propaedeutic, “Laikon” General Hospital, Athens Medical School, 75 Micras Asias Street, Goudi, 11527 Athens, Greece; ^2^1st Pathology Laboratory, Athens Medical School, 75 Micras Asias Street, Goudi, 11527 Athens, Greece; ^3^Radiotherapy Unit, 2nd Radiology Department, “Attikon” University General Hospital, Athens Medical School, Rimini 1, Xaidari, 12462 Athens, Greece; ^4^2nd Pathology Laboratory, “Attikon” University General Hospital, Athens Medical School, Rimini 1, Xaidari, 12462 Athens, Greece

## Abstract

*Background*. Inflammation mediators related to radiation proctitis are partially elucidated, and neovascularization is thought to play a key role. *Objectives*. To investigate the expression of vascular endothelial growth factor (VEGF) and CD31 as angiogenetic markers in postradiation rectal tissue. *Methods*. Rectal mucosa biopsies from 11 patients who underwent irradiation for prostate cancer were examined immunohistochemically for the expression of VEGF and CD31 at three time settings—before, at the completion of, and 6 months after radiotherapy. VEGF expressing vascular endothelial cells and CD31 expressing microvessels were counted separately in 10 high-power fields (HPFs). VEGF vascular index (VEGF-VI) and microvascular density (MVD) were calculated as the mean number of VEGF positive cells per vessel or the mean number of vessels per HPF, respectively. Histological features were also evaluated. *Results*. VEGF-VI was significantly higher at the completion of radiotherapy (0.17 ± 0.15 versus 0.41 ± 0.24, *P* = 0.001) declining 6 months after. MVD increased significantly only 6 months after radiotherapy (7.3 ± 3.2 versus 10.5 ± 3.1, *P* < 0.005). The histopathological examination revealed inflammatory changes at the completion of radiotherapy regressing in the majority of cases 6 months after. *Conclusions*. Our results showed that in postradiation rectal biopsy specimens neoangiogenesis seems to be inflammation-related and constitutes a significant postradiation component of the tissue injury.

## 1. Introduction

Irradiation is an important adjuvant therapy in the treatment of pelvic malignancies. However, it often results in collateral damage to the surrounding of the primary tumor site. The most frequent complication after radiotherapy treatment for prostate cancer is radiation proctitis with incidence rates ranging from 2% to 39% [1]. Radiation damage may occur in acute or chronic form. Acute radiation proctitis occurs immediately or up to 3 months after the initiation of radiotherapy. The presenting symptoms are diarrhea, tenesmus, urgency, mucus discharge, and bloody stools. In contrast, chronic proctitis may appear years after the completion of therapy usually manifesting by gross hemorrhage. The newer irradiation modalities—3D conformal radiotherapy and intensity-modulated radiotherapy—are usually not associated with severe side effects such as ulceration, fistulation, necrosis, and stricture, but bleeding due to radiation proctitis still occurs even at a lower rate. Radiation proctitis is diagnosed by endoscopy where edematous, friable, and with abnormal telangiectatic vessels mucosa is usually demonstrated [1, 2]. 

The pathophysiology of radiation proctitis is only partially elucidated, and several mechanisms have been put forward. The earliest studies suggested that blood vessels are the main site of injury and that microvascular compromise is an important factor in the natural history of radiation proctitis. The pathogenetic process triggered by radiation seems to be multifactorial including several molecular events leading to inflammation, hypoxia, neovascularization, and fibrosis [3–5]. Various cytokines and growth factors have been implicated in the pathogenesis including hypoxia-inducible factor 1 (HIF-1), transforming growth factor *β*1 (TGF-*β*1), fibroblast growth factor 1 (FGF-1), and vascular endothelial growth factor (VEGF) [6–8]. Radiation-induced inflammatory response is closely related to oxidant stress, while an increase in free oxygen radical production has been documented as a result of infiltrating inflammatory cells and of radiation-induced ischemia [9, 10].

Angiogenesis plays an important role in many chronic inflammatory diseases, while VEGF is the main stimulatory factor [11]. It is secreted by macrophages, endothelial cells, fibroblasts, smooth muscle cells, and activated platelets. VGEF induces proliferation, inhibits apoptosis of endothelial cells, increases vascular permeability, and has a chemotactic effect on macrophages. VEGF gene expression is regulated by various mechanisms the most important being hypoxia, especially through upregulation of HIF [12, 13]. 

There is very limited and conflicting experimental data regarding the role of angiogenesis in the context of postradiation proctitis, especially in the chronic form of the disease. The information derives mainly from mice studies where a gradual increase of VEGF after an early peak of HIF was observed in rectal tissues during the first 3 months after their irradiation [6]. As for humans, an increased angiogenesis not corresponding to a VEGF overexpression has been demonstrated in cases of radiation proctitis at 7–38 months after irradiation [7]. 

The aim of our study was the immunohistochemical investigation of angiogenesis in postradiation rectal mucosa in association with VEGF expression and in relation to histological findings in the early and late postradiation period in order to provide some evidence regarding the involvement of neoangiogenesis in mucosal injury.

## 2. Materials and Methods

### 2.1. Sample Selection

We prospectively enrolled consecutive patients with prostate cancer who were newly referred and treated with 3D conformal radiotherapy schedule of 72–74 Gy of total dose. In all patients, the total dose did not exceed 70 Gy for more than 25% of rectal volume. Four rectal biopsies were obtained randomly at sigmoidoscopy from normal-appearing mucosa at least 1 cm away from any macroscopically-visible lesion using a 6 mm forceps. The endoscopy was performed before, at the completion of (within an interval of 1–3 days), and 6 months after radiotherapy. None of the individuals had a personal history of colorectal cancer, and all had the same bowel preparation. The study was approved by the hospital's ethics committee, and an informed consent was signed by every individual before the procedures.

### 2.2. Histology and Immunohistochemistry

All biopsy specimens were fixed in formalin solution and processed according to routine protocol. Four *μ*m thick paraffin sections were stained with hematoxylin and eosin for histological assessment and for immunohistochemical analysis. The following primary antibodies were applied: (i) monoclonal mouse anti-human CD31 antibody (MO823; Dako, Glostrup, Denmark) at a dilution 1 : 50 and (ii) monoclonal mouse anti-human VEGF antibody (M7273; Dako, Glostrup, Denmark) at a dilution 1 : 50. Detection was carried out using the DakoEnvision Detection System Peroxidase/DAB+ (K4065; Dako, Glostrup, Denmark).

### 2.3. Histological Examination and Assessment of Immunostaining

Histological evaluation included inflammatory infiltrates, presence or absence of cryptitis and of crypt abscesses, erosions or ulceration, and thickening of the subepithelial collagen plate. 

For the evaluation of immunostaining, 10 high-power fields were examined arbitrarily under ×400 magnification for each case. VEGF positive vascular endothelial cells and CD31 expressing microvessels were counted. VEGF “vascular index” (VEGF-VI) and “microvascular density” (MVD), that is, the mean number of VEGF positive cells per vessel or the mean number of vessels per field, were calculated, respectively. The vessels with staining of CD31 were counted to examine MVD. Average of all fields was used for the analysis of VEGF and MVD. Immunohistochemical assessment was performed by two examiners, blinded to clinical information. For each case, agreement was reached by simultaneous evaluation of the specimens using a two-headed microscope. 

### 2.4. Statistical Analysis

Statistical analysis was performed using the statistical package Statgraphics Centurion XV (StatPoint Technologies, Inc. Corp. Warrenton, VA, USA). Results in the text are presented as mean with standard deviation. The paired *t*-test was used to compare differences between groups. A *P* value <0.05 was considered significant. Data in figures are presented as box-and-whisker plots. The box includes 50% of the results falling between the 25th and 75th percentile (interquartile distance). The median value is represented as a horizontal line inside the box. Outliers, that is, points more than 1.5 times the interquartile range from the end of the box, are shown as open squares.

## 3. Results

Eleven patients with a mean age of 72.4 ± 10.5 years were studied. Three additional patients missed to followup and were excluded from the analysis. During the followup period, 2 patients reported rectal bleeding that settled without specific intervention, while in 4 patients endoscopy revealed the presence of mild to moderate radiation proctitis not needing a therapeutic intervention according to endoscopic classification by Zinicola et al. [14].

### 3.1. Histology

Preirradiation biopsies showed normal rectal mucosa in all 11 patients. Histological findings in the early postirradiation period were predominantly characterized by inflammatory changes. In 6 cases, inflammatory changes were consisted with mild active colitis characterized by infiltration of the lamina propria mainly by neutrophils, mild cryptitis, and few crypt abscesses. Small telangiectasias were found only in one case, while fibrin microthrombi were not observed.  Table 1 summarized the histological findings in an individual patient at the completion of radiotherapy.

**Figure 1:**
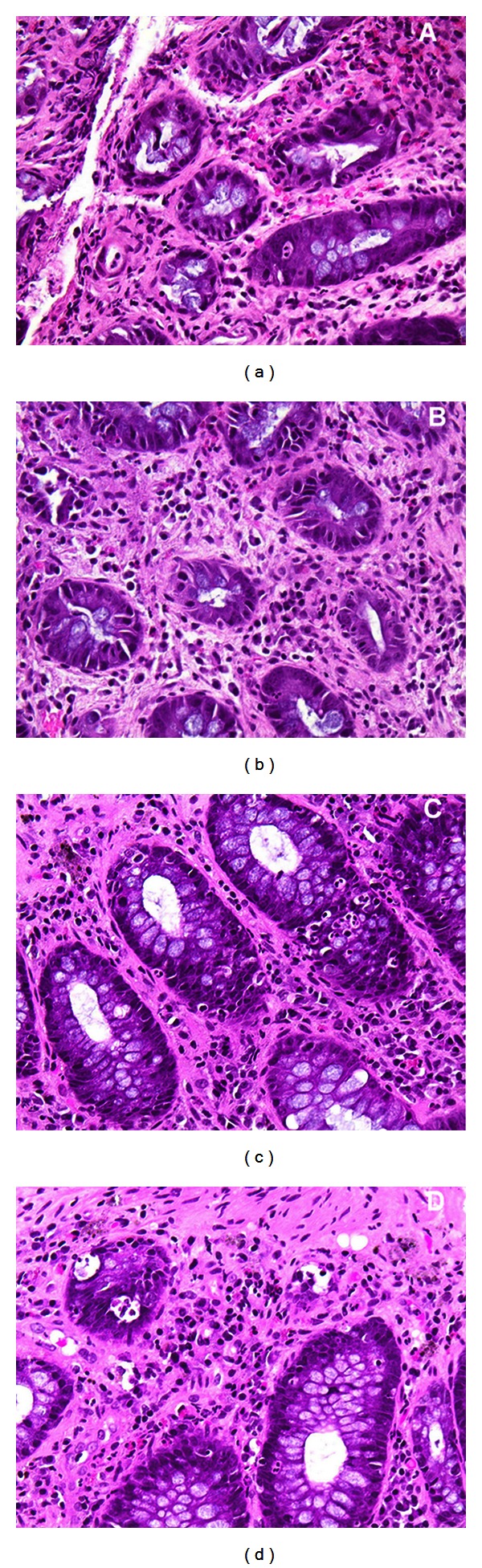
Representative inflammatory epithelial changes in rectal tissue in the early postirradiation period consisting of infiltration of the lamina propria by neutrophils, cryptitis, and few crypt abscesses (c, d). Additional findings were mild focal fibrosis (a–d), mild crypt distortion, and atrophy (a, b).

In the late postirradiation period, the predominant diagnosis was mild nonspecific chronic colitis which was ascribed to 8 cases.  Table 1 summarized the histological findings in an individual patient 6 months after completion of radiotherapy. The typical histological changes are presented in Figure 1. Histological findings such as crypt distortion, fibrosis, and vascular telangiectasia were limited probably due to the fact that our study was restricted to the “early” postirradiation period (first 6 months). Moreover, radiation tissue damage is expected to be less severe in relation to the contemporary radiation modalities.

### 3.2. Immunohistochemistry

**Figure 2:**
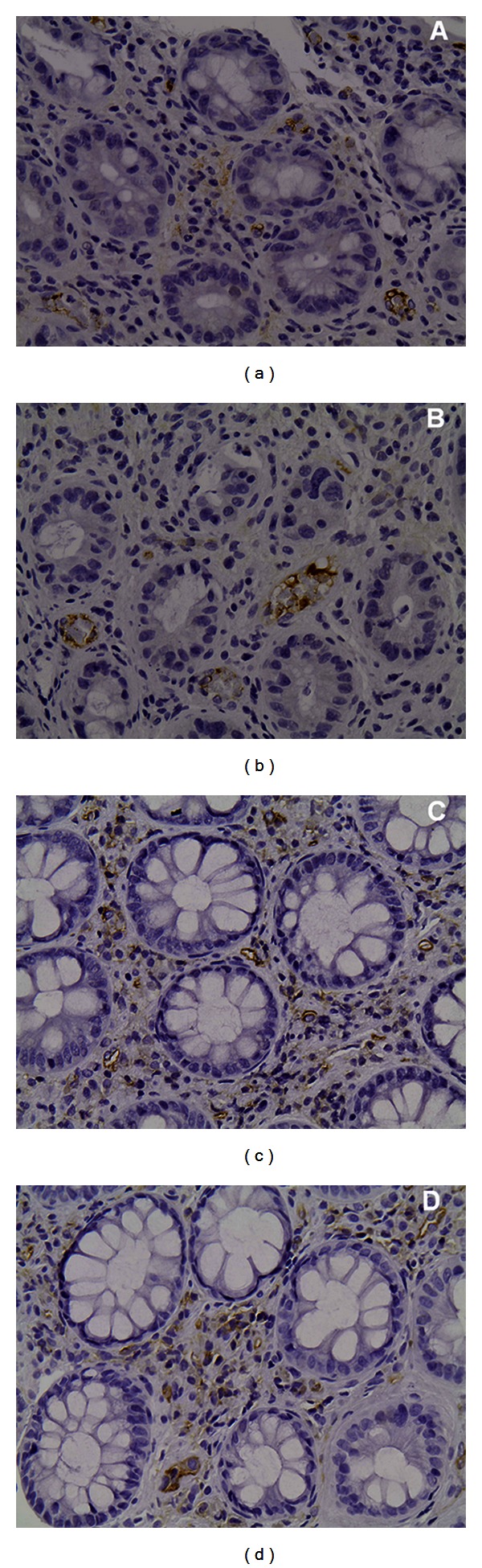
Rectal mucosa immunostained (a, b) with anti-VEGF antibody (M7273; Dako, Glostrup, Denmark) and (c, d) with anti-CD31 antibody (MO823; Dako, Glostrup, Denmark). Microvasculature was demonstrated by CD31 immunostaining of the vascular endothelial cells while VEGF was detected in endothelial cells and in few stroma cells showing cytoplasmic staining.

Microvasculature was demonstrated by CD31 immunostaining, while VEGF was detected in endothelial cells and in few stroma cells showing cytoplasmic staining (Figure 2). Both VEGF-VI and MVD were increased at the completion of radiotherapy, the difference being significant only for VEGF-VI; 0.41 ± 0.24 versus 0.17 ± 0.15, *P* = 0.001 and 7.6 ± 2.2 versus 7.3 ± 3.2,  *P* = 0.61, respectively. At the time of completion of radiotherapy, the mean values of VEGF and CD31 were significantly higher in cases with active colitis in histology examination compared to those showing no activity (0.55 ± 0.12 versus 0.24 ± 0.23, *P* = 0.012 and 8.95 ± 1.9 versus 6.08 ± 1.43, *P* = 0.02, resp.). 

**Figure 3:**
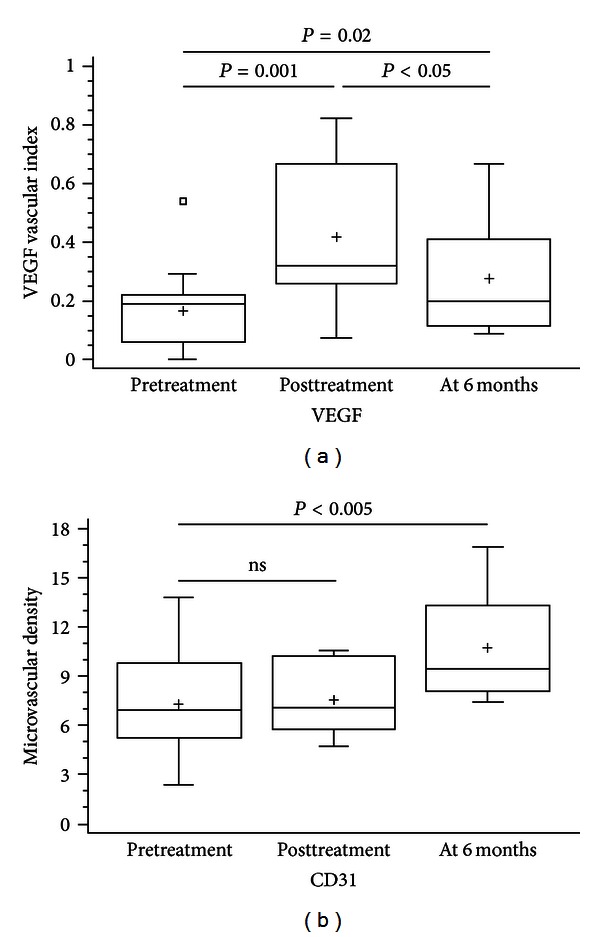
Evaluation of immunostaining for vascular endothelial growth factor (VEGF vascular index) and CD31 (microvascular density) in rectal mucosa of patients with radiation proctitis due to radiotherapy for prostate cancer at three time settings. VEGF vascular index (a) was significantly increased at the completion of irradiation and remained so, at 6 months. In contrary, microvascular density (b) was unchanged at the completion of radiotherapy but increased significantly at 6 months.

The increases of both indexes were also observed six months after the end of radiotherapy; the difference was significant for VEGF-VI and MVD compared to those before irradiation (0.29 ± 0.19 versus 0.17 ± 0.15, *P* = 0.02 and 10.5 ± 3.1 versus 7.3 ± 3.2, *P* < 0.005, resp.). VEGF-VI six months after the end of radiotherapy was significantly lower compared to index at the time of radiotherapy completion (0.29 ± 0.19 versus 0.41 ± 0.24, *P* < 0.05). The results are summarized in Figure 3, while Table 2 presented the sequential change of VEGF and CD31 in an individual patient. 

At the completion of the radiotherapy, there was a trend, which did not reach significance, for correlation between VEGF and CD31 (*P* = 0.087). The same, without statistical significance, trend was also observed after 6 months of radiotherapy completion (*P* = 0.099). 

## 4. Discussion

Radiotherapy has been established as the treatment of choice for patients with prostate cancer. The use of conformal radiotherapy succeeded in reducing irradiation to organs at risk such as rectum, enabling a higher dose to the target volume [15, 16]. Even with this advanced technique, symptoms suggestive of postradiation rectum damage occur in up to 20% of patients depending on the dose and treatment method [17–19]. Acute symptoms observed early after external beam therapy of prostate cancer are thought to be mainly inflammatory in nature and settled spontaneously over few months after exposure in the majority of patients. However, in selected individuals postradiation reactions could be sustained for unclear reasons for months and even years after radiotherapy. This chronic form of radiation proctitis seems to be the result of submucosal inflammation, fibrosis, and angiogenesis [20]. 

Angiogenesis is a complex process mediated by multiple cells types and mediators, and besides its well-known role in cancer, it plays a critical role in hypoxic conditions and in several chronic inflammatory diseases [21]. Moreover, it potentiates the inflammatory response by increasing the influx of inflammatory cells as well as a chemotactic mediator [22, 23]. In our study, histological examination revealed an increased vascularity in rectal mucosa six months after radiation exposure. It was preceded by mucosal inflammation and concomitant VEGF expression appearing as early changes shortly after irradiation, while neither acute nor chronic ischemic lesions were found. These consecutive findings provide indications of a pathogenetic link between inflammation and vascularization, taking into consideration the higher values of VEGF and CD31 expression in patients with active colitis at the end of radiotherapy. The occurrence and/or persistence of newly formed microvessels after remission both of the inflammatory process and of the decrease of VEGF expression suggest a later postradiation and postinflammatory manifestation. Since now there are only few treatment options for patients with symptoms, such as bleeding due to radiation proctitis. Endoscopic treatment with argon plasma coagulation (APC) is considered the preferred treatment modality for radiation proctitis. Although APC successfully ameliorates symptoms associated with mild endoscopic radiation proctitis, it is less effective in severe cases of the disorder. In these cases, intrarectal formalin—nevertheless an absolute toxic agent—is a useful therapeutic strategy [14, 24, 25]. According to our results, angiogenesis constitutes a component of mucosal injury in radiation proctitis; the clinical significance of new vessels formation following VGEF expression relies on the putative higher risk of bleeding complications. Thus, we could speculate on a possible effectiveness of antiangiogenetic drugs regarding the inhibition of excessive vascularization and the reduction of bleeding complications. However, the use of these compounds is restricted to cancer treatment and has been only experimentally investigated in colitis models and in IBD patients [26]. 

Our findings in the early postradiation period have demonstrated an association of increased VEGF expression with radiation-induced inflammation probably related to oxidative stress. This observation favors a beneficial impact of anti-inflammatory and antioxidant medication early in the course of postirradiation proctitis by preventing the initiation of inflammatory process directly after irradiation. Although someone could argue that there is no need to treat asymptomatic proctitis, it is well known to clinicians that asymptomatic radiation-induced proctitis is potentially a symptomatic one with or without rectal bleeding with an increasing time-related possibility. Thus, radiation-proctitis should be treated in a prevention manner, and our results showing early involvement of VGEF in the pathogenesis of this disease imply that the blockage of this factor could be a promising therapeutic option. Under this view, a combination of vitamin E (400 IU *tid*) and vitamin C (500 mg *tid*) has been proved a successful and sustained treatment of chronic radiation proctitis [27]. 

A limitation of our study is the fact that due to the relatively small study populations, we were not able to make any correlation among clinical symptoms, endoscopic findings, and microscopic features. As only a minority of our patients had symptoms (2 had bleeding that settled without specific intervention) or endoscopic finding of radiation proctitis (4 patients), further studies are needed in order to examine a putative relation between histological findings and clinical symptoms. Moreover, the period that we choose to evaluate our patients could raise concerns, as late radiation-induced rectal injury might occur months or years after radiotherapy. We evaluated our patients with endoscopy 6 months after radiotherapy completion because our objective was to identify factors of prognostic significance regarding the course of the disease. Early proctoscopy, even in asymptomatic but endoscopically confirmed rectal damage, has a significant role in predicting late radiation-induced proctitis [28, 29].

In conclusion, our study showed that in postradiation rectal biopsy specimens neoangiogenesis seems to be inflammation-related and constitutes a significant postradiation component of the tissue injury. The involvement of inflammation meditator VEGF in the pathogenesis of radiation proctitis suggests that the blockage of the expression of this factor may represent a promising therapeutic option in patients with refractory to available therapies cases of the disorder. 

## Figures and Tables

**Table 1:** Histological findings in an individual patient at three time settings—before, at the completion of, and 6 months after radiotherapy.

	Before radiotherapy	At the completion of radiotherapy	6 months after radiotherapy
Patient 1	Normal	Mild active colitis-mild focal crypt atrophy-focal fibrosis	Mild nonspecific chronic inflammation
Patient 2	Normal	Focal fibrosis-mild crypt distortion	Mild nonspecific chronic inflammation-mucinophages
Patient 3	Normal	Focal fibrosis-mild focal crypt atrophy-thickening of the subepithelial collagen band	Mild nonspecific chronic inflammation-mild focal fibrosis-mucinophages
Patient 4	Normal	Focal fibrosis-mild focal crypt atrophy-thickening of the subepithelial collagen band	Mild nonspecific chronic inflammation
Patient 5	Normal	Mild active colitis	Mild nonspecific chronic inflammation-few mucinophages-focal fibrosis
Patient 6	Normal	Mild active colitis	Mild nonspecific chronic inflammation
Patient 7	Normal	Mild active colitis-few angiectasis	Mild nonspecific chronic inflammation
Patient 8	Normal	Mild active colitis-focal fibrosis	Thickening of the subepithelial collagen band-few mucinophages
Patient 9	Normal	Mild active colitis-thickening of the subepithelial collagen band	Focal crypt atrophy
Patient 10	Normal	Focal fibrosis-mild focal crypt atrophy-mild crypt distortion	Mild nonspecific chronic inflammation-few mucinophages
Patient 11	Normal	Mild crypt distortion	Focal fibrosis-thickening of the subepithelial collagen band

**Table 2:** Sequential change of VEGF and CD31 in an individual patient at three time settings—before, at the completion of, and 6 months after radiotherapy.

Patient	CD31_pre_radio	CD31_at completion	CD31_6 months after	VEGF_pre_radio	VEGF_at completion	VEGF_6 month after
1	7.7	7.2	4.5	0.37	0.59	0.38
2	6.5	4.3	9.1	0.13	0.63	0.52
3	12.8	5.2	5.6	0.42	0.05	0.10
4	7.3	7.8	9.3	0.41	0.26	0.46
5	2.4	7.5	11.3	0.10	0.64	0.58
6	3.6	9.2	12.7	0.10	0.48	0.44
7	7.5	10.3	11.7	0.10	0.65	0.18
8	5.3	11.9	13.2	0.08	0.62	0.16
9	7.9	7.6	11.7	0.08	0.34	0.12
10	11.7	7.2	12.3	0.06	0.04	0.10
11	10.6	5.9	14.3	0.05	0.23	0.10
